# Prevalence of and factors associated with homebound status among adults in urban and rural Spanish populations

**DOI:** 10.1186/s12889-016-3270-z

**Published:** 2016-07-15

**Authors:** Laureano Negrón-Blanco, Jesús de Pedro-Cuesta, Javier Almazán, Carmen Rodríguez-Blázquez, Esther Franco, Javier Damián, on behalf of the DISCAP-ARAGON Research Group

**Affiliations:** Xeral-Calde Hospital Complex, Lucus Augusti University Teaching Hospital, Lugo, Spain; Department of Applied Epidemiology, National Centre for Epidemiology (Carlos III Institute of Health), Madrid, Spain; Consortium for Biomedical Research in Neurodegenerative Diseases (Centro de Investigación Biomédica en Red sobre Enfermedades Neurodegenerativas/CIBERNED), Madrid, Spain; Faculty of Medicine & Health Sciences, School of Physiatry and Nursing, Zaragoza University, Zaragoza, Spain; Centro Nacional de Epidemiología (Instituto de Salud Carlos III), Av. de Monforte de Lemos 5, 28029 Madrid Spain

**Keywords:** Homebound status, Disability, Dependence, Older people, Ageing

## Abstract

**Background:**

There is a marked growth in the number of homebound older adults, due mainly to increased life expectancy. Although this group has special characteristics and needs, it has not been properly studied. This study thus aimed to measure the prevalence of homebound status in a community-dwelling population, and its association with both socio-demographic, medical and functional characteristics and the use of health care and social services.

**Methods:**

We used instruments coming under the WHO International Classification of Functioning (ICF) framework to carry out a cross-sectional study on populations aged 50 years and over in the province of Zaragoza (Spain), covering a total of 1622 participants. Persons who reported severe or extreme difficulty in getting out of the house in the last 30 days were deemed to be homebound. We studied associations between homebound status and several relevant variables in a group of 790 subjects who tested positive to the WHODAS-12 disability screening tool.

**Results:**

Prevalence of homebound status was 9.8 % (95 % CI: 8.4 to 11.3 %). Homebound participants tended to be older, female and display a lower educational level, a higher number of diseases, poorer cognition and a higher degree of disability. In fully adjusted models including disability as measured with the ICF-Checklist, the associated variables (odds ratios and [95 % confidence intervals]) were: female gender (3.75 [2.10–6.68]); urban population (2.36 [1.30–4.29]); WHODAS-12 disability (6.27 [2.56–15.40]); depressive symptoms (2.95 [1.86–4.68]); moderate pain (2.37 [1.30–4.31] and severe pain (3.03 [1.31–7.01]), as compared to the group with no/mild pain; hospital admissions in the previous 3 months (2.98 [1.25–7.11]); and diabetes (1.87 [1.03–3.41]). Adjustment for ICF-Checklist disability had a notable impact on most associations.

**Conclusions:**

The study shows that homebound status is a common problem in our setting, and that being disabled is its main determinant. Socio-demographic characteristics, barriers and chronic diseases can also be assumed to be playing a role in the onset of this condition, indicating the need for further research, including longitudinal studies on its incidence and associated factors.

## Background

Homebound status is an activity limitation, estimated by various studies to have a prevalence in the general population of 1.6 % to 4.7 %, which may rise to 60 % in people over 85 years and is higher in rural areas and among women [1–4].

There is wide variation in reported prevalences of this disability, probably due to the lack of a standard criterion to define it, as well as differences in study populations in terms of age range and social and demographic conditions [1, 2, 5]. Epidemiological studies tend to use self-reported criteria to define this limitation, such as subjects’ need of help from another person to get out of the house or the frequency with which they do so. Qiu et al. conducted a review of the criteria used by different authors to define homebound status [6]. The most appreciable differences were found in the period of time spent without leaving home, which ranged from going out once per week or less [3], to those who responded “never or almost never except in cases of emergency”, when asked how often they went out for any reason [7].

Homebound status has been found to be associated with different variables that favour and perpetuate it, and with increases in health care costs and greater caregiver burden [1, 8, 9]. The main related variables are age, degree of cognitive impairment, dependence in basic and instrumental activities of daily living (ADL), depression, loss of visual acuity, obesity and low body weight, number of comorbidities, educational level, living in a multi-storey building with no lift, and not owning a car. Difficulty in leaving the home is viewed by these studies as a predictive factor of mortality and disability, after adjustment for demographic variables, and pathological, functional and psychosocial processes [10–12].

Our bibliographic review found no studies that characterised homebound status on the basis of probabilistic samples in Spain. Some primary care studies on groups of patients included in home-care programmes have focused on assessing the functional and cognitive status and quality of life of the users of such programmes [13, 14].

In view of what is known about the association between the rise in life expectancy [15] and the progressive increase in the incidence of chronic diseases, functional loss, and other conditions shown to generate difficulty in persons in terms of mobility and travel outside the home, we felt it necessary to conduct a descriptive study with a wide number of variables, so as to have a tool when it came to undertaking other specific studies, planning and managing health service policies, and to act as a point of departure for possible interventions on potentially modifiable factors. The aim of this study was thus to estimate the prevalence of and factors associated with homebound status in an urban and rural population of community-dwelling persons aged 50 years and over.

## Methods

The study data were drawn from a survey conducted from 2008 to 2011. The sampling frame comprised health-card holders aged 50 years and older, residing in the Zaragozan district of Cinco Villas, and in two health areas of the city of Zaragoza. In 2006, public health-care coverage in Aragon was 98.7 % and per capita GDP was 10 % higher than that for Spain as a whole [16].

### Participant selection

We initially selected 2000 subjects from Cinco Villas and 856 from the city of Zaragoza by simple random sampling. From this sample, some individuals were then excluded for any of the following reasons: (i) subject was not a resident in the study area (317 Cinco Villas; 15 Zaragoza); (ii) subject could not be located (222 Cinco Villas; 5 Zaragoza); (iii) subject had died (101 Cinco Villas; 1 Zaragoza); and, (iv) subject refused to participate or was unable to schedule or keep appointment for evaluation purposes (110 Cinco Villas; 330 Zaragoza). From Cinco Villas, 48 subjects were excluded due to lack of sufficient data, leaving a net study sample of 1707 individuals with complete evaluations, 1202 from Cinco Villas and 505 from Zaragoza: from these we excluded institutionalised persons, which left a total of 1633 for study purposes, 1135 from Cinco Villas and 498 from Zaragoza.

Information on most variables was obtained via personal interviews with subjects or close relatives (ICF Checklist sores being obtained by professional assessors). Informed consent was obtained from patients and information was provided to patients’ primary care physicians, as described by de Pedro-Cuesta et al. [17]. Data were collected in two stages, in accordance with a screening scheme. Data on socio-demographic characteristics (sex, age, marital status, living arrangements and education) and cognitive status were collected for the entire sample*,* and individuals with a minimum degree of disability were screened, using the WHODAS 12-item scale (Appendix), a shortened version of the disability assessment tool recommended by the World Health Organisation (WHO) for epidemiological studies, namely, the 36-item World Health Organisation Disability Assessment Schedule, WHODAS II (WHODAS-36) [18]. These instruments are validated scales with good psychometric properties, as shown by evaluation with both classical and item response methods [19–21]. Subjects with 1 or more points on the WHODAS-12 were deemed to be positive to screening, and underwent a thorough disability assessment, with information also being collected on medical history, depressive symptoms, quality of life, use of services, and other variables described below. Those with a *Miniexamen Cognoscitivo* (MEC) score of less than 24 points (see below) were likewise deemed to be positive to screening. The MEC is the Spanish version of the Mini-mental State Examination, and has been adequately validated for use on the Spanish population [22].

### Dependent variable: homebound status

For case-definition purposes, we used the following WHODAS-36 question, “*In the past 30 days, how much difficulty did you have in leaving home? None/mild/moderate/severe/extreme or cannot do*”, with a response of “severe” or “extreme or cannot do” being construed as “homebound”. This question was not part of WHODAS-12 (See Appendix). The question was posed to individuals who tested positive to disability screening, and was answered by these respondents or their caregivers. Those who tested negative to screening (0 points on the WHODAS-12) were deemed to be “non-homebound”.

### Habits

Detailed data were collected on the frequency of consumption of alcoholic beverages in the preceding year, and converted into “standard drink units” per day [23]. Information on tobacco use was obtained through detailed interviews, and subjects were then grouped into never smokers, ex-smokers (those who had smoked in the past but who were not smoking at the time of the interview) and current smokers.

### Anthropometric variables

We measured weight and height (one measurement), and obtained body mass index, categorised as low or normal weight (<25 kg/m^2^), overweight (≥25-29.9 kg/m^2^) or obesity (≥30 kg/m^2^).

### Cognitive status

An MEC score <24was deemed to be indicative of cognitive impairment.

### Disability as limitation of activity and restriction on participation

The following two instruments were used: the dichotomous WHODAS-12 (Appendix) to measure the association between disability and homebound status, with the cut-point for disability set at ≥20 points; and the International Classification of Functioning, Disability and Health (ICF) Checklist [24]. This amounts to a comprehensive evaluation of aspects of disability by purpose-trained assessors. Among other aspects, the ICF Checklist assesses ability to perform a series of activities (“limitations on activity and restriction on participation” domain). Scores were allocated according to the degree of difficulty experienced in each activity, on a scale of 0 (no disability) to 100 (maximum disability). This score was used as a control variable in certain analyses. In addition to disability, we also assessed dependence in the performance of basic ADL, using the Katz Index [25] (for a fuller review of the functional assessment of this population, see de Pedro-Cuesta et al. [17]).

### Chronic diseases

In the Cinco Villas district, diagnoses were generally obtained from primary care clinical records by direct manual examination by trained field workers (health professionals). At the two Zaragoza centres, diagnoses were obtained from electronic records. Diagnostic data were completed during the home visit. In a few cases, where participants had no medical records at primary care centres, data or hospital discharge reports were furnished by relatives. We drew up a list of 22 chronic diseases and subsequently added 2 more, corresponding to vision and audition impairments, which included any patient registering severe or complete impairment in the “impairments of body functions” section of the ICF Checklist. We recorded the occurrence of these 24 possible conditions, and their number.

### Self-rated health and depressive symptoms

Self-rated health was measured using the EQ-5D visual analogue scale [26], on which subjects rated their current health from 0 (worst health status possible) to 100 (best health status possible), with the cut-point set at ≤50. Depressive symptoms were assessed using the 12 questions of the EURO-D scale [27, 28], range 0 to 12 points, with the cut-point for possible depression set at ≥4 [28].

### Use of health care services and caregiver support

Subjects or close relatives were asked about participants’ use of services in the preceding three months, including nursing services, visits to (or from) the primary care physician and/or specialist, rehabilitation services, and hospital admissions and re-admissions. Subjects were also asked about the presence of caregivers, both professional and non-professional.

### Pain

Based on the assessment obtained in the “impairments of body functions” section of the ICF Checklist, participants were grouped into 3 categories, i.e., “no/mild”, “moderate” or “severe”.

### Data-analysis

Overall prevalence of homebound status was calculated as the number of homebound cases over the total study population. The association between homebound status and different variables was measured using logistic regression models. A first group of models included age group (50–64, 65–79, ≥80 years), sex, years of education and sampling area (rural, comprising Cinco Villas district, and urban, comprising the city of Zaragoza). A second group of models added: degree of disability as per the Activities & Participation domain of the ICF Checklist (the score was included in the logarithmic transformation); cognitive status (dichotomous MEC); and number of chronic disorders. Possible effect modification by the variable disability, under the Activities & Participation domain of the ICF Checklist, was then studied by creating two groups, including those with no or mild disability (0 to 24 points) and those with moderate-to-severe disability (25 to 100 points). Effects were calculated by including interaction terms between the ICF Checklist disability dichotomous variable and the variables of interest in the models fitted. All data analyses were performed using the Stata version 13.1 statistical software programme (Stata Corp., College Station, Texas).

## Results

The overall response rate was 60 % (1707/2856). Of the 1707 persons, 74 were excluded due to being institutionalised and a further 11 for lacking valid data on homebound status, leaving a total study population of 1622. As compared to the study population, the excluded group (1149) had a lower proportion of women (47.4 % vs. 56.7.0 %) and a similar mean age (68.4 y vs. 68.3 y). Of the 1622 study subjects, 159 were considered homebound, yielding a homebound population prevalence among persons aged 50 years and over of 9.8 % (95 % CI: 8.4 %–11.3 %). The prevalence (95 % CI) of homebound status was 10.0 % (8.4 %–11.9 %) for CincoVillas and 9.3 % (7.0 %–12.2 %) for Zaragoza. Table 1 shows the crude data on the population sample of 1622 individuals, which was characterised by a higher proportion of women and a higher age among those who were homebound, though the most pronounced differences were seen in terms of cognitive impairment and disability.

**Table 1:** Basic characteristics of participants according to homebound status in the complete sample: percentages, mean and associational odds ratio

Variable	Non-homebound *N* (%)	Homebound *N* (%)	OR (95 % CI)	*P-*value
Total	1463 (100)	159 (100)		
Sex, women	794 (54.3)	126 (79.2)	3.02 (1.96–4.64)	<0.001
Age group, years				<0.001
50–64	648 (44.3)	15 (9.4)	1 (ref.)	
65–79	611 (41.7)	49 (30.8)	3.46 (1.92–6.24)	
≥80	204 (14.0)	95 (59.7)	20.12 (11.41–35.46)	
Marital status, with spouse	1045 (71.6)	74 (46.8)	0.78 (0.52–1.16)	<0.001
Living arrangements, living alone	214 (14.6)	17 (10.8)	0.35 (0.19–0.63)	0.19
Educational level				<0.001
Less than primary	459 (31.3)	94 (59.1)	1 (ref.)	
Primary	664 (45.4)	51 (32.1)	0.38 (0.26–0.54)	
Secondary or higher	340 (23.2)	14 (8.8)	0.20 (0.11–0.36)	
Cognitive impairment (MEC <24)	57 (3.9)	70 (46.7)	9.01 (5.49–14.78)	<0.001
Disability (WHODAS 12)				
Mean (SD)	14.7 (5.5)	33.6 (9.4)	18.9^a^ (17.9–19.9)	<0.001
Moderate or severe (≥20 points)	255 (17.3)	152 (95.6)	69.20 (29.89–160.24)	<0.001

*SD* standard deviation, *MEC Miniexamen Cognoscitivo*, *OR* Odds ratio, *WHODAS* World Health Organisation Disability Assessment Schedule

^a^Mean difference and 95 % confidence interval

Table 2 shows the results for the population that tested positive to disability screening, comprising 790 persons with valid data for homebound status. This table shows associations with two levels of adjustment. At the less complex level, note should be taken of the associations with female gender (OR = 2.38), age (ORs of 1.93 and 6.39 for the two groups aged over 65 years versus that aged 50–64 years), number of chronic disorders (ORs of 2.40 and 4.09 for groups of 2 to 3, and 4 or more chronic disorders, respectively), cognitive impairment (OR = 5.56), disability as measured with the WHODAS 12 scale (OR = 28.23) or with the ICF-Checklist (OR = 18.17), and dependence in at least 1 ADL (OR = 6.77). Presence of a caregiver displayed a strong association (OR = 7.40). In terms of self-perceived variables, the associations were clear, in the case of both self-rated health (OR = 4.02) and depressive symptoms (OR = 2.95). Lastly a strong association was observed with pain, though this was rated by assessors. When adjustment was made for the number of chronic disorders, ICF-Checklist disability score and cognitive impairment, the previous associations were maintained, though some changed notably, even in direction. The practically non-existent association between rural/urban areas became very clear, with the greatest risk of home confinement being observed in urban settings (OR = 2.36). While the effect of age disappeared, that of disability remained pronounced, though there was a clear weakening (from OR = 28.23 to OR = 6.27). The important effect of dependence in ADL and caregiver presence disappeared (Table 2).

**Table 2:** Association between selected variables and homebound status in a population testing positive to disability screening

Variable	*N* (%)	Homebound *n* (%)	OR (95 % CI)^a^	OR (95 % CI)^b^
All	790 (100)	159 (20)		
Population, urban	219 (28)	46 (21)	1.23 (0.80–1.87)	2.36 (1.30–4.29)
Sex, women	519 (66)	126 (24)	2.38 (1.52–3.73)	3.75 (2.10–6.68)
Age group, years				
50–64	210 (27)	15 (7)	1 (ref).	1 (ref).
65–79	335 (42)	49 (15)	1.93 (1.03–3.60)	0.66 (0.30–1.45)
≥80	245 (31)	95 (39)	6.39 (3.44–11.84)	0.63 (0.28–1.43)
Marital status, with spouse	489 (62)	74 (15)	0.81 (0.54–1.22)	0.97 (0.57–1.65)
Educational level				
Less than primary	343 (43)	94 (27)	1 (ref).	1 (ref).
Primary	344 (44)	51 (15)	0.62 (0.41–0.95)	0.94 (0.54–1.61)
Secondary or higher	103 (13)	14 (14)	0.95 (0.48–1.90)	1.93 (0.77–4.85)
Living arrangements, alone	129 (16)	17 (13)	0.37 (0.20–0.66)	0.88 (0.45–1.74)
Tobacco use				
Never smoker	532 (68)	131 (25)	1 (ref).	1 (ref).
Ex-smoker	174 (22)	21 (12)	0.71 (0.36–1.39)	0.79 (0.32–1.93)
Current smoker	81 (10)	5 (6)	0.54 (0.18–1.65)	0.61 (0.16–2.31)
Alcohol consumption (≥1 standard drink/day)	241 (31)	13 (5)	0.25 (0.13–0.47)	0.56 (0.27–1.16)
Body Mass Index (kg/m^2^)				
<25	150 (22)	31 (21)	1 (ref).	1 (ref).
25–29.9	293 (43)	44 (15)	0.75 (0.42–1.32)	0.80 (0.38–1.64)
≥30	239 (35)	49 (21)	1.16 (0.66–2.04)	1.01 (0.49–2.06)
Number of chronic disorders				
0–1	225 (29)	16 (7)	1 (ref).	1 (ref).
2–3	381 (48)	79 (21)	2.40 (1.31–4.41)	2.28 (0.85–6.06)
≥4	180 (23)	64 (36)	4.09 (2.13–7.82)	4.32 (0.83–22.50)
Cognitive impairment (MEC <24)	127 (16)	70 (55)	5.56 (3.41–9.09)	0.94 (0.49–1.80)
Disability (WHODAS 12 ≥ 20)	407 (52)	152 (37)	28.2 (12.2–65.4)	6.27 (2.56–15.40)
ICF Checklist disability, moderate/severe	230 (26)	122 (53)	18.2 (10.9–30.2)	10.45 (5.99–18.23)
Dependence in ≥1 basic ADL	317 (40)	128 (40)	6.77 (4.23–10.82)	1.18 (0.65–2.12)
Nursing services	517 (66)	115 (22)	1.04 (0.67–1.63)	0.97 (0.55–1.68)
Visit, primary care physician	613 (78)	117 (19)	0.73 (0.46–1.14)	0.81 (0.44–1.48)
Visit, specialist physician	399 (51)	84 (21)	1.43 (0.96–2.12)	1.76 (1.03–3.00)
Hospital admissions	55 (7)	23 (42)	4.04 (2.05–7.95)	2.98 (1.25–7.11)
Rehabilitation services	62 (8)	11 (18)	1.17 (0.55–2.47)	0.88 (0.35–2.22)
Use of a caregiver	226 (29)	109 (48)	7.40 (4.72–11.6)	0.74 (0.39–1.40)
Self-rated health, less than good (≤50)	343 (56)	78 (24)	4.02 (2.21–7.32)	2.02 (0.99–4.11)
Depressive symptoms (EURO-D ≥4)	226 (30)	60 (26)	2.95 (1.86–4.68)	2.31 (1.34–3.99)
Pain				
No/mild	352 (45)	46 (13)	1 (ref).	1 (ref).
Moderate	370 (47)	79 (21)	1.78 (1.14–2.77)	2.37 (1.30–4.31)
Severe	59 (8)	25 (42)	5.91 (2.93–11.9)	3.03 (1.31–7.01)

*ADL* activities of daily living, *CI* confidence interval, *MEC Miniexamen cognoscitivo*, *OR* odds ratio, *WHODAS* World Health Organisation Disability Assessment Schedule

^a^Adjusted for sex, age group, area (rural/urban), and number of years of formal education

^b^Additionally adjusted for number of chronic disorders, disability (ICF Checklist score, in logarithmic transformation), and cognitive impairment (yes/no)

Table 3 shows the associations between homebound status and different chronic conditions, especially cerebrovascular disease (OR = 2.69), COPD (OR = 2.23), diabetes (OR = 2.30), cancer (OR = 2.13), depression (OR = 2.03), dementia (OR = 3.14), severe mental disease (OR = 7.09), neurodegenerative diseases and dystrophies (OR = 3.62), and visual (OR = 3.06) and audition impairments (OR = 2.35). While the additional adjustment diluted most of these associations, the association with diabetes nonetheless persisted (OR = 1.87).

**Table 3:** Association between chronic disorders and homebound status in a population testing positive to disability screening

Disease	Disease prevalence *N* (%)	OR (95 % CI)^a^	OR (95 % CI)^b^
Hypertension	372 (47)	0.71 (0.48–1.06)	0.86 (0.52–1.43)
Ischaemic heart disease	68 (9)	0.94 (0.47–1.88)	1.18 (0.49–2.82)
Arrhythmias	102 (13)	1.16 (0.69–1.96)	0.87 (0.44–1.74)
Heart failure	25 (3)	1.55 (0.61–3.95)	1.04 (0.32–3.38)
Cerebrovascular disease	86 (11)	2.69 (1.56–4.64)	0.84 (0.41–1.70)
Peripheral arterial disease	14 (2)	0.74 (0.15–3.67)	0.57 (0.09–3.42)
COPD	62 (8)	2.23 (1.16–4.29)	1.73 (0.78–3.84)
Asthma	25 (3)	0.95 (0.33–2.77)	0.56 (0.14–2.27)
Diabetes	134 (17)	2.30 (1.44–3.68)	1.87 (1.03–3.41)
Thyroid diseases	75 (9)	0.87 (0.45–1.67)	0.90 (0.37–2.19)
Chronic renal failure	29 (4)	2.94 (1.28–6.77)	1.39 (0.51–3.77)
Chronic liver disease	6 (1)	1.01 (0.10–10.22)	2.55 (0.22–28.96)
Anaemia	33 (4)	0.81 (0.31–2.11)	0.68 (0.22–2.09)
Cancer	42 (5)	2.13 (0.96–4.76)	1.94 (0.68–5.51)
Anxiety disorder	69 (9)	0.49 (0.22–1.10)	0.56 (0.22–1.45)
Depression	140 (18)	2.03 (1.28–3.22)	1.40 (0.78–2.49)
Dementia	47 (6)	3.14 (1.60–6.16)	0.24 (0.09–0.65)
Neurodegenerative diseases and dystrophies^c^	17 (2)	3.62 (1.16–11.33)	1.24 (0.30–5.06)
Severe mental disease	8 (1)	7.09 (1.35–37.16)	0.46 (0.05–4.15)
Arthritis or arthrosis	401 (51)	0.96 (0.65–1.41)	1.79 (1.05–3.05)
Hip fracture	13 (2)	2.19 (0.59–8.13)	1.21 (0.17–8.80)
Visual impairment	56 (7)	3.06 (1.64–5.73)	1.53 (0.68–3.46)
Audition impairment	36 (5)	2.35 (1.09–5.07)	1.46 (0.56–3.79)
Urinary incontinence	42 (5)	1.45 (0.71–2.97)	0.64 (0.26–1.59)

*COPD* chronic obstructive pulmonary disease, *CI* confidence interval, *OR* odds ratio

^a^Adjusted for sex, age group, area (rural/urban), and number of years of formal education

^b^Additionally adjusted for number of chronic disorders (excluding index disease), disability (ICF Checklist score, in logarithmic transformation), and cognitive impairment (yes/no) except in dementia

^c^Parkinson’s disease, Multiple Sclerosis and Amyotrophic Lateral Sclerosis

Exploratory analyses, not shown, revealed that the great changes in effects seen in the fully adjusted models in Tables 2 and 3 were essentially due to the inclusion of the variable disability in the Activities & Participation domain of the ICF Checklist. It thus became necessary to explore the possible modification of effect exerted by this variable, i.e., whether estimates would be different depending on ICF-Checklist disability status. These analyses are shown in Table 4. Differences were observed in specialist medical visits (*p*-value for interaction = 0.03), arthritis (*p* = 0.03), and also likely in the case of history of depression (*p* = 0.08), with all of these displaying a higher association with confinement in the group without ICF-Checklist disability. On the other hand, the association between age and homebound status was seen solely in the group with ICF-Checklist disability (*p* = 0.03), suggesting a synergistic effect of both variables. In addition, homebound status appeared to be associated with hospital admission only in patients with disability (OR = 4.00 vs. OR = 0.95 in the non-disability group, *p*-value for interaction = 0.03). Some estimates in Table 4 should be interpreted with caution, due to high random error.

**Table 4:** Association between selected variables and homebound status according to ICF Checklist degree of disability

	No/mild		Moderate/severe		
Variable	*N* (%)^a^	OR (95 % CI)^b^	*N* (%)^a^	OR (95 % CI)^b^	*P* ^c^
Population, urban	13 (8)	0.99 (0.49–2.03)	33 (67)	1.75 (0.84–3.65)	0.27
Sex, women	32 (8)	2.83 (1.08–7.43)	94 (67)	3.36 (1.79–6.29)	0.77
Age group, years					0.03
50–64	11 (6)	1 (ref.)	4 (27)	1 (ref.)	
65–79	14 (5)	0.66 (0.28–1.55)	35 (52)	3.54 (0.95–13.12)	
≥ 80	12 (12)	1.64 (0.66–4.05)	83 (58)	2.47 (0.71–8.60)	
Marital status, with spouse	20 (5)	0.64 (0.32–1.30)	54 (52)	1.37 (0.72–2.61)	0.11
Educational level					0.13
Less than primary	18 (9)	1 (ref.)	76 (52)	1 (ref.)	
Primary	13 (5)	0.54 (0.25–1.16)	38 (59)	1.52 (0.77–3.00)	
Secondary or higher	6 (7)	0.92 (0.33–2.58)	8 (50)	1.68 (0.49–5.82)	
Living arrangements, alone	7 (7)	0.72 (0.30–1.74)	10 (38)	0.50 (0.19–1.27)	0.57
Tobacco use					0.19
Never smoker	30 (8)	1 (ref.)	101 (63)	1 (ref.)	
Ex-smoker	2 (2)	0.33 (0.07–1.46)	19 (35)	0.91 (0.37–2.26)	
Current smoker	4 (6)	0.81 (0.22–2.98)	1 (10)	0.17 (0.02–1.58)	
Alcohol consumption (≥1 standard drink/day)	5 (2)	0.39 (0.14–1.03)	8 (22)	0.37 (0.15–0.93)	0.96
Body mass index (kg/m^2^)					0.23
<25	6 (6)	1 (ref.)	25 (50)	1 (ref.)	
25–29.9	13 (6)	0.96 (0.34–2.69)	31 (48)	0.72 (0.30–1.74)	
≥30	17 (10)	1.81 (0.67–4.89)	32 (50)	0.64 (0.27–1.55)	
No. of chronic disorders					0.18
0–1	4 (2)	1 (ref.)	12 (44)	1 (ref.)	
2–3	22 (8)	4.61 (1.32–16.09)	57 (49)	1.18 (0.39–3.61)	
≥ 4	11 (11)	7.37 (1.25–43.45)	53 (65)	2.56 (0.46–14.15)	
Cognitive impairment (MEC <24)	3 (14)	2.31 (0.62–8.67)	67 (64)	2.28 (1.23–4.22)	0.99
Dependence in ≥ 1 basic ADL	19 (13)	2.91 (1.43–5.95)	109 (63)	2.94 (1.31–6.58)	0.99
Nursing services	23 (6)	0.71 (0.33–1.54)	92 (57)	1.30 (0.67–2.51)	0.23
Visit, primary care physician	32 (7)	1.62 (0.55–4.75)	85 (53)	0.77 (0.39–1.51)	0.25
Visit, specialist physician	29 (10)	3.60 (1.53–8.46)	55 (56)	1.13 (0.61–2.11)	0.03
Hospital admissions	1 (4)	0.95 (0.12–7.62)	22 (73)	4.00 (1.40–11.41)	0.23
Rehabilitation services	4 (9)	1.30 (0.43–3.90)	7 (41)	0.80 (0.23–2.71)	0.56
Use of a caregiver	8 (15)	2.83 (1.18–6.81)	101 (59)	1.93 (0.94–3.94)	0.50
Self-rated health, less than good (≤50)	24 (9)	3.45 (1.43–8.35)	54 (60)	3.17 (1.17–8.59)	0.90
Depressive symptoms (EURO-D ≥4)	20 (13)	3.52 (1.68–7.34)	40 (61)	2.42 (1.18–4.94)	0.47
Pain					0.21
None/mild	6 (2)	1 (ref.)	40 (40)	1 (ref.)	
Moderate	25 (9)	4.41 (1.65–11.80)	54 (59)	1.53 (0.78–3.00)	
Severe	6 (18)	11.5 (3.17–41.3)	19 (76)	4.24 (1.39–12.94)	
Hypertension	16 (6)	0.77 (0.38–1.54)	56 (49)	0.71 (0.38–1.33)	0.88
Ischaemic heart disease	2 (5)	0.93 (0.21–4.15)	15 (50)	0.89 (0.35–2.24)	0.96
Arrhythmias	1 (2)	0.21 (0.03–1.58)	28 (61)	1.32 (0.61–2.87)	0.09
Heart failure	2 (17)	2.07 (0.43–9.87)	7 (54)	1.02 (0.28–3.72)	0.49
Cerebrovascular disease	2 (6)	0.95 (0.21–4.23)	31 (58)	1.64 (0.77–3.48)	0.53
Peripheral arterial disease	0 (0)	^d^	2 (33)	0.48 (0.07–3.15)	
COPD	1 (3)	0.52 (0.06–4.10)	19 (63)	2.36 (0.97–5.75)	0.19
Asthma	0 (0)	^d^	6 (55)	0.82 (0.20–3.41)	
Diabetes	10 (12)	2.47 (1.12–5.48)	31 (65)	1.92 (0.89–4.14)	0.65
Thyroid diseases	5 (9)	1.24 (0.46–3.37)	10 (56)	0.59 (0.19–1.80)	0.33
Chronic renal failure	3 (33)	5.21 (1.18–23.02)	11 (55)	1.39 (0.49–3.97)	0.15
Chronic liver disease	1 (20)	3.31 (0.34–32.15)	0 (0)	^d^	
Anaemia	1 (5)	0.85 (0.11–6.63)	5 (38)	0.43 (0.12–1.53)	0.58
Cancer	1 (4)	0.71 (0.09–5.56)	9 (64)	3.28 (0.84–12.85)	0.22
Anxiety disorder	4 (7)	0.96 (0.32–2.89)	4 (31)	0.26 (0.07–0.93)	0.13
Depression	14 (15)	2.85 (1.36–5.95)	29 (60)	1.15 (0.56–2.37)	0.08
Dementia	0 (0)	^d^	26 (60)	0.68 (0.29–1.59)	
Neurodegenerative diseases and dystrophies^e^	2 (33)	16.6 (2.64–105.0)	5 (45)	0.96 (0.23–4.05)	0.02
Severe mental disease	0 (0)	^d^	4 (67)	1.48 (0.21–10.51)	
Arthritis or arthrosis	29 (10)	3.10 (1.37–7.02)	61 (56)	0.99 (0.54–1.82)	0.03
Hip fracture	1 (20)	3.51 (0.36–34.05)	6 (75)	1.76 (0.23–13.33)	0.66
Visual impairment	2 (9)	1.67 (0.36–7.76)	24 (73)	3.23 (1.30–7.98)	0.47
Audition impairment	0 (0)	^d^	16 (62)	2.20 (0.82–5.86)	
Urinary incontinence	3 (14)	1.75 (0.47–6.44)	13 (62)	0.72 (0.27–1.94)	0.29

*ADL* activities of daily living, *COPD* chronic obstructive pulmonary disease, *CI* confidence interval, *ICF* international classification of functioning, *MEC Miniexamen cognoscitivo*, *OR* odds ratio, *WHODAS* World Health Organisation Disability Assessment Schedule

^a^Number and percentage of homebound cases

^b^Odds ratios calculated by means of logistic regression models that included interaction terms between the dichotomous variable “disability” (in the Activities & Participation domain of the ICF Checklist) and the study variables. The models also included sex, age group, area (rural/urban), and number of years of formal schooling (except in educational level), chronic disorders (excluding index disease), and cognitive impairment (yes/no) except in dementia

^c^ *P*-value for interaction

^d^Empty cells due to lack of subjects in relevant subgroup

^e^Parkinson’s disease, Multiple Sclerosis and Amyotrophic Lateral Sclerosis

## Discussion

In view of the characteristics of our data, this study on homebound status was approached with purely descriptive and enumerative purposes in mind, with no intention of testing hypotheses of causality. Its principal limitation lay in not having measures of all the variables of disability, morbidity and use of socio-health services for the general population, with our analysis of associations being limited to the group that tested positive to disability screening, though it has to be said here that the screening level is very low, inasmuch as any person having a minimal indication of disability tests positive (see WHODAS-12 scale in Appendix). Despite being a very sensitive test, false negatives cannot be excluded (homebound subjects not captured into the screening), though such a possibility is regarded as extremely unlikely.

One factor to be borne in mind is that, being a cross-sectional design, the directionality of the associations cannot be properly evaluated. Some associations might possibly be bidirectional, e.g., depression may play a role in the occurrence of confinement and conversely, confinement may lead to depression. Hence, the context should be considered when interpreting each particular estimate.

Although the overall non-response rate was 40 %, this was essentially for administrative reasons. There was a higher-than-expected proportion of women in the study population and no difference in mean age. Even so, this difference can be expected to have had little impact on the overall prevalence of homebound status, since neither the difference in the female to male sex ratio nor the difference in the non-response rate was excessive. As regards gender and age, non-response had a negligible impact on the associational estimates because all were adjusted for both variables.

### Prevalence

Prevalence of homebound status is within the range reported in other series. The crude data showed no relevant differences in prevalence between urban and rural areas, with this only becoming evident after multivariate adjustment, as in other studies [29].

### Socio-demographic profile

Our study confirms the association between age and homebound status, accounted for in great part by multimorbidity and related disability. The effect of age disappears when disability is adjusted for (Table 2). Confronted with this unusual result, the possibility of interaction was examined, and a protective age effect was found in the group with least disability and an effect of increased risk in the group with disability, which practically amounts to cancelling out the joint effect. Prior studies in groups attended by primary home care services, likewise show an association between home confinement and advanced age, with this being more prevalent in the female sex [14, 30]. The marked sex-related difference found is essentially attributable to the greater life expectancy of women with severe disability, as well as the higher female to male sex ratio in the older age strata, in which individuals are more susceptible both to homebound status and to disability and multimorbidity. This may also be related to social differences that place women at least risk of institutionalisation, as documented in earlier studies [29, 31, 32].

### Morbidity

Other population-based studies have described how different chronic disorders and the number of such disorders are associated with difficulty in leaving the home. A review by Qui et al. reported an association with the presence, both of chronic metabolic, cardiovascular, cerebrovascular and locomotor system disorders, and of cognitive impairment, dementia and depression [6]. Our study found an association with the number of chronic diseases and with history of dementia, depression, cerebrovascular and neurodegenerative diseases, chronic obstructive pulmonary disease (COPD), diabetes, severe visual and audition impairments, urinary incontinence and osteoarticular disease. It should be noted that when adjustment is made for disability, these associations disappear, which suggests that disability is the intermediate step between the latter variables and homebound status. Stress should be laid on the clear association with diabetes, a result that has been found by other studies to be a predictor of risk of disability due to multiple physiopathological mechanisms and this disease’s association with other, mainly cardiovascular comorbidities [33–35].

In our population, homebound status was associated with disability assessed by the WHODAS-12 and ICF Checklist, and with dependence in basic ADL (Katz Index). Previous studies in the same or other populations found disability and comorbidity to be the principal factors linked to difficulty in leaving the home, a situation equally related to worse self-rated health and depression [28, 36–38].

### Use of health care services

In our sample, we found a positive association between the condition under study and hospital admissions and readmissions in the preceding three months, as well as a greater use of specialised ambulatory medical services, contrary to what could be expected in terms of the difficulties faced in moving outside the home and gaining access to public transport. Other studies have reported a higher risk of hospital admissions in this population sector [39].

## Conclusions

The study shows that homebound status is a common problem in our setting. Socio-demographic characteristics, barriers, chronic diseases and functional status can be assumed to be playing a role in the occurrence of this condition, indicating the need to conduct longitudinal studies on its incidence and associated factors.

## Abbreviations

ADL, activities of daily living; COPD, chronic obstructive pulmonary disease; ICF, international classification of functioning; MEC, *Miniexamen Cognoscitivo* (Spanish version of the Mini-mental State Examination); WHODAS, World Health Organisation Disability Assessment Schedule

## Appendix

### 12-item World Health Organisation disability assessment scale (WHODAS 12)

*Range*: 0 to 48 points.

1. How much difficulty did you have in standing for long periods such as 30 min?
  *None (0 points); Mild (1 point); Moderate (2 points); Severe (3 points); Extreme/Cannot do (4 points).*
2. How much difficulty did you have in taking care of your household responsibilities?
3. How much difficulty did you have in learning a new task, for example, learning how to get to a new place?
4. How much of a problem did you have joining in community activities (for example, festivities, religious or other activities) in the same way as anyone else can?
5. How much have you been emotionally affected by your health problems
6. How much difficulty did you have in concentrating on doing something for ten minutes?
7. How much difficulty did you have walking a long distance such as a kilometre [or equivalent]?
8. How much difficulty did you have in washing your whole body?
9. How much difficulty did you have in getting dressed?
10. How much difficulty did you have in dealing with people you do not know?
11. How much difficulty did you have in maintaining a friendship?
12. How much difficulty did you have in your day-to-day work?
